# Effect of Doping TiO_2_ NPs with Lanthanides (La, Ce and Eu) on the Adsorption and Photodegradation of Cyanide—A Comparative Study

**DOI:** 10.3390/nano13061068

**Published:** 2023-03-16

**Authors:** Ximena Jaramillo-Fierro, Ricardo León

**Affiliations:** 1 Departamento de Química, Facultad de Ciencias Exactas y Naturales, Universidad Técnica Particular de Loja, San Cayetano Alto, Loja 1101608, Ecuador; 2 Maestría en Química Aplicada, Facultad de Ciencias Exactas y Naturales, Universidad Técnica Particular de Loja, San Cayetano Alto, Loja 1101608, Ecuador

**Keywords:** cyanide, adsorption, photocatalysis, anatase, lanthanide doping, nanoparticles

## Abstract

Free cyanide is a highly dangerous compound for health and the environment, so treatment of cyanide-contaminated water is extremely important. In the present study, TiO_2_, La/TiO_2_, Ce/TiO_2_, and Eu/TiO_2_ nanoparticles were synthesized to assess their ability to remove free cyanide from aqueous solutions. Nanoparticles synthesized through the sol–gel method were characterized by X-ray powder diffractometry (XRD), scanning electron microscopy (SEM), energy-dispersive X-ray spectroscopy (EDS), Fourier-transformed infrared spectroscopy (FTIR), diffuse reflectance spectroscopy (DRS), and specific surface area (SSA). Langmuir and Freundlich isotherm models were utilized to fit the adsorption equilibrium experimental data, and pseudo-first-order, pseudo-second-order, and intraparticle diffusion models were used to fit the adsorption kinetics experimental data. Cyanide photodegradation and the effect of reactive oxygen species (ROS) on the photocatalytic process were investigated under simulated solar light. Finally, reuse of the nanoparticles in five consecutive treatment cycles was determined. The results showed that La/TiO_2_ has the highest percentage of cyanide removal (98%), followed by Ce/TiO_2_ (92%), Eu/TiO_2_ (90%), and TiO_2_ (88%). From these results, it is suggested that La, Ce, and Eu dopants can improve the properties of TiO_2_ as well as its ability to remove cyanide species from aqueous solutions.

## 1. Introduction

Cyanides are highly toxic chemical compounds that can cause severe harm to health and the environment [1]. The most common cyanide compounds in the environment are present both in their free form, which comprises the cyanide ion itself (CN^−^) and hydrogen cyanide (HCN), and as water-soluble inorganic salts, including sodium cyanide (NaCN) and potassium cyanide (KCN) [2]. Some cyanide compounds are naturally produced by microorganisms, although they can also be found in plants and some foods, as well as in low concentrations in soil and water. On the other hand, generation of effluents by industries is the main source of release into the environment of cyanide complexes, which are formed by union of cyanide ions with metal ions [3].

All forms of cyanide can be lethal at high levels of exposure; however, free cyanide (HCN + CN^−^) is the deadliest form of all [4]. This form of cyanide in a solution can be removed and/or transformed to fewer toxic forms through various physicochemical methods, such as acidification, photocatalysis, adsorption, coagulation, reverse osmosis, precipitation, filtration, and AVR (acidification, volatilization, and reneutralization) process [5,6]. Among these methods, adsorption and photocatalysis have shown to be two promising methodologies due to their high efficiency, simplicity of design, ease of handling, and low operating cost [7,8]. The adsorption process can be carried out by different mechanisms, including electrostatic interaction, ion exchange, and complexation [9]. Unfortunately, this process only allows for transfer of the contaminant from one medium to another without completely removing it from the environment. However, complete degradation of the adsorbed pollutant can be achieved through the series of oxidation–reduction reactions that take place in the photocatalytic process [10,11,12,13].

Photocatalysis applied to aqueous or gaseous systems involves formation of reactive oxygen species (ROS), such as superoxide (^•^O_2_^−^), hydroxyl radical (^•^OH), and singlet oxygen (^1^O_2_), which are produced by electron capture by oxygen or oxidation of water molecules [2]. ROS are very effective oxidizing agents, capable of degrading recalcitrant compounds, and can also be produced on the surface of semiconductor materials, such as titanium dioxide (TiO_2_) [14]. TiO_2_ is widely used as a photocatalyst because it is cheap, abundant, chemically inactive, and photostable to corrosion; it also has high thermal stability, intense photocatalytic activity, strong oxidizing power, and is friendly to the environment [15]. TiO_2_ can produce ROS when it accepts a minimum quantity of energy (bandgap energy, E_g_) that allows it to remove an electron from the valence band (VB) and transport it to the conduction band (CB), thus generating an electron/hollow (e^−^/h^+^) [16]. Unfortunately, the bandgap energy (E_g_ = 3.2 eV) required by TiO_2_ for pair formation (e^−^/h^+^) restricts use of this semiconductor to wavelengths in the UV region (≈ 390 nm), which represents only 5% of solar light [17]. Another important limitation of TiO_2_ is its high recombination of photogenerated charges, which diminishes formation of ROS and, consequently, its photocatalytic efficiency [18].

In order to decrease recombination of the pairs (e^−^/h^+^) and shift the absorption wavelength to the wanted visible region (>λ = 400 nm), several specific surface modifications on TiO_2_ have been suggested, including doping with different elements, including lanthanides [19,20,21,22,23]. In fact, lanthanide metal ions (Ln^3+^) have been employed as TiO_2_ dopants in numerous investigations since they can considerably modify the physicochemical properties of this photocatalyst [24]. Doping of TiO_2_ with lanthanide elements can occur through two mechanisms. In one mechanism, elements can be incorporated in the TiO_2_ lattice by direct bonding or substitution to produce a ≡Ti–O–Ln–O–Ti≡ arrangement, causing lattice defects/distortions due to the difference in ionic radii of Ln^3+^ and Ti^4+^. In the other mechanism, it is considered that there is not enough energy to promote ion substitution in the lattice, so the lanthanide elements can disperse on the TiO_2_ surface, creating Ti–O–Ln bonds [25]. Presence of lanthanides, scattered as surface impurities in the TiO_2_ structure, causes vacancies and surface defects that enable capture of the electrons produced on the surface of the photocatalyst, thus reducing recombination of photogenerated charge carriers [26]. However, an excess of lanthanide dopants on the TiO_2_ surface can reduce its photocatalytic capacity, possibly due to generation of elevated density of defects and vacancies, which would behave as recombination centers and not as electron collectors [27].

Lanthanide elements have motivated numerous investigations due to their potential industrial applications, derived from their exceptional optical, magnetic, and/or redox properties. Lanthanides, in particular lanthanum (La), cerium (Ce), and europium (Eu), when applied as TiO_2_ dopants, inhibit transition from the anatase phase to the rutile phase, cause lattice distortions in the surface layer, generate defects that reduce crystallite size, increase specific surface area, improve thermal stability, increase the number of oxygen vacancies, readily react with organic compounds to mineralize them, and can also provide luminescent properties to TiO_2_ [28,29]. Lanthanum, cerium, and europium have been studied for their capacity to enhance the photocatalytic activity of TiO_2_, probably due to the presence of two events: a decrease in their bandgap energy and generation of an imbalance in their surface charges [30,31]. Both events lead to states that contribute to capture of photogenerated electrons and reduce recombination of pairs (e^−^/h^+^), increasing the probability of formation of hydroxyl radicals (^•^OH) that are essential for photodegradation of organic molecules [32]. Presence of these elements on the TiO_2_ surface leads to generation of Lewis acid sites that ensure catalytic stability of semiconductors in aqueous reaction media [25,33]. Likewise, Lewis acid sites enable increasing the adsorption of contaminant molecules on the TiO_2_ surface, which improves transfer of electrons for direct degradation of the pollutant and increases the possibility of interaction between contaminant molecules and photogenerated radicals [34].

As can be seen, doping with lanthanide elements has demonstrated to be effective in adapting the TiO_2_ surface to several applications [35]. However, almost surprisingly, no investigations have been found in the literature on use of TiO_2_ doped with La, Ce, or Eu for adsorption and photocatalytic degradation of total cyanide in aqueous systems. Therefore, the objective of this comparative study is to evaluate the effect of La, Ce, and Eu ions on the adsorbent and photocatalytic capacity of TiO_2_ for its application in removal of total cyanide from aqueous solutions. Thus, in the present study, adsorption and photocatalysis experiments were designed in batch reactors, where the amount of residual total cyanide (HCN + CN^−^) in the solutions was determined by UV–visible spectrophotometry using the picrate alkaline method. The adsorption capacity of the synthesized nanoparticles was evaluated by modifying the composition of the doping element, the pH of the solutions, the initial concentration of the adsorbate, and the contact time, while the photocatalytic activity was determined by simulated solar irradiation (λ = 300–800 nm). The synthesized nanoparticles (TiO_2_, La/TiO_2_, Ce/TiO_2_, and Eu/TiO_2_) were characterized by X-ray powder diffractometry (XRD), Fourier-transformed infrared spectroscopy (FTIR), diffuse reflectance spectroscopy (DRS), scanning electron microscopy (SEM), energy-dispersive X-ray spectroscopy (EDS), and specific surface area (SSA) by the BET method. 

The novelty of this comparative study lies in the fact that it was possible to demonstrate that doping of TiO_2_ with lanthanide elements, particularly Lanthanum, enables efficient removal of cyanide species from aqueous systems at neutral pH thanks to the effective combination of the processes of adsorption and photocatalysis. The materials synthesized in this study could represent an important innovation in environmental technology due to their operability and reusability, thus providing a sustainable alternative with great application potential in effluent treatment.

## 2. Materials and Methods

### 2.1. Materials

The following reagents (analytical grade) were used in the present investigation without any further purification: Europium(III) nitrate pentahydrate (Eu(NO_3_)_3_∙5H_2_O, Sigma Aldrich, St. Louis, MO, USA, 99.9%), Cerium(III) nitrate hexahydrate (Ce(NO_3_)_3_∙6H_2_O, Sigma Aldrich, St. Louis, MO, USA, 99.9%), Lanthanum nitrate hexahydrate (La(NO_3_)_3_∙6H_2_O, Sigma Aldrich, St. Louis, MO, USA, 99.9%), Titanium (IV) isopropoxide (Ti(OC_3_H_7_)_4_, Sigma Aldrich, St. Louis, MO, USA, 98.0%), Isopropyl alcohol (C_3_H_8_O, Sigma Aldrich, St. Louis, MO, USA, ≥99.5%), Hydrochloric acid (HCl, Sigma Aldrich, St. Louis, MO, USA, 37.0%), Sodium hydroxide (NaOH, Sigma Aldrich, St. Louis, MO, USA, ≥85.0%), Potassium cyanide (KCN, Sigma Aldrich, St. Louis, MO, USA, ≥97.0%), Picric acid ((O_2_N)_3_C_6_H_2_OH, Sigma Aldrich, St. Louis, MO, USA, ≥99.0%), Sodium carbonate (Na_2_CO_3_, Sigma Aldrich, St. Louis, MO, ≥99.0%), *p*-Benzoquinone (C_6_H_4_(=O)_2_, Sigma Aldrich, St. Louis, MO, USA, ≥98.0%), Ethanol (CH_3_CH_2_OH, Sigma Aldrich, St. Louis, MO, USA, 95.0%).

### 2.2. Synthesis of the Nanoparticles

The TiO_2_ (TO), La/TiO_2_ (La/TO), Ce/TiO_2_ (Ce/TO), and Eu/TiO_2_ (Eu/TO) nanoparticles were synthesized following an adapted sol–gel method explained in previous studies [36]. To obtain the TiO_2_ nanoparticles, a solution (Solution A) of titanium (IV) isopropoxide (TiPO) dissolved in isopropyl alcohol (iPrOH) at a TiPO/iPrOH ratio of 70% *v/v* was prepared at room temperature. A solution (Solution B) of isopropyl alcohol (iPrOH), iPrOH/water (50% *v/v*) was also prepared at room temperature. Solution B was gradually added dropwise to solution A, maintaining constant stirring and room temperature. After formation of a white precipitate, the reaction system was stirred for 60 min at room temperature. The precipitate was dried (60 °C) for 24 h and then calcined (500 °C) for 4 h. Finally, the obtained solids were cooled to room temperature. To achieve the La/TO, Ce/TO, and Eu/TO doped nanoparticles, the procedure described above was repeated, adding the lanthanum, cerium, or europium salts to the aqueous solution (Solution B) to achieve a final concentration of the doping element of ~1% per gram of TiO_2_.

### 2.3. Characterization of the Nanoparticles

Characterization of the nanoparticles was performed using the methodology described in our previous study [36]. For X-ray diffraction (XRD) measurements, a Bruker-AXS D8-Discover diffractometer (Bruker AXS, Karlsruhe, Germany) was used. For specific surface area (SSA) determination, ChemiSorb 2720 equipment (Micromeritics, Norcross, GA, USA) was used. Micrographs of the synthesized samples were obtained by field effect scanning electron microscopy (SEM) on a Zeiss Gemini ULTRA plus electron microscope (Carl Zeiss AG, Ober-kochen, Germany). A Phenom ProX (Phenom World BV, Eindhoven, The Netherlands) was used to achieve energy-dispersive X-ray (EDS) spectra. In the present study, quantification of the elements in the synthesized nanoparticles was also performed by inductively coupled plasma (ICP) optical emission spectrometry (OES) in an Optima 8000 ICP-OES Spectrometer (PerkinElmer, Inc., Waltham, MA, USA). FTIR spectra were recorded on a PerkinElmer GX2000-FTIR Spectrometer (PerkinElmer, Inc., Waltham, MA, USA). The diffuse UV–vis reflectance spectra (DRS) were obtained using a Phenom DesNicolet Evolution 201/220 Thermo UV–vis spectrophotometer (ThermoFisher, Waltham, MA, USA). To simulate solar light and evaluate the photoactivity of the nanoparticles, a solar box ATLAS, SUNTEST CPS+ (Atlas Material Testing Technology, Mount Prospect, IL, USA) was used. Finally, the amount of cyanide remaining in the solutions was quantified using a Jenway 7350 spectrophotometer (Cole-Parmer, Staffordshire, UK). IBM SPSS (version 25.0; statistic software for Windows; IBM Corp.; Armonk, NY, USA, 2017) was used for the ANOVA analysis. The crystalline phases were recognized using the ICDD database (International Center for Diffraction Data, version 2018). Finally, the Chemisoft TPx system (version 1.03; data analysis software; Micromeritics, Norcross, GA, USA, 2011) was used to calculate the SSA by the single-point method using the Brunauer–Emmet–Teller (BET) equation. 

### 2.4. Adsorption Studies

Cyanide adsorption experiments from KCN aqueous solutions were designed to evaluate the following aspects: (a) the effect of pH on cyanide adsorption, (b) maximum cyanide adsorption capacity, (c) adsorption thermodynamics and (d) the kinetic behavior of cyanide adsorption. The data obtained from the adsorption experiments were fitted to isothermal and kinetic models using the least squares nonlinear regression method [37]. Experiments were performed with all nanoparticles using the methodology described in our previous study [36]. Cyanide species adsorption experiments were performed using a batch method at room temperature and keeping the pH of the solutions at 7.0 ± 0.1 by adding 0.1 M solutions of hydrochloric acid or sodium hydroxide. The amount of catalyst used in all the experiments was 0.2 g L^−1^. The maximum cyanide adsorption capacity was investigated by varying the concentration of 500 mL of KCN solution from 0.20 to 40 mg L^−1^. The adsorption thermodynamics and kinetic behavior of cyanide adsorption were investigated using 500 mL of water containing 20 mg L^−1^ of KCN [38]. The alkaline picrate analytical method was used to quantify total cyanide in aqueous solutions [39,40,41]. To accomplish this, first, the alkaline picrate solution (PAS) was prepared, consisting of 1 g of picric acid ((O_2_N)_3_C_6_H_2_OH) and 5 g of sodium carbonate (Na_2_CO_3_) dissolved in 200 mL of HPLC water. A volume of 4 mL of this solution was added to 1 mL of cyanide or blank solution (HPLC water) contained in a test tube. This tube was incubated for 5 min in a 95 °C water bath. After this time, the absorbance at 490 nm was measured using a UV–vis spectrophotometer. The concentration of the solutions was determined based on the previously prepared calibration curve (R^2^ = 0.9996) according to the Lambert–Beer Law. The tests were carried out in triplicate and the results were expressed as the average of three repetitions [38]. The amount of cyanide adsorbed (q_e_) on the nanoparticles expressed in mg g^−1^ was estimated using the following equation [42]:

(1) $$q_{e}=\left(C_{0}-C_{e}\right)\times \frac{v}{w}$$

where C_0_ and C_e_ are expressed in mg L^−1^ and correspond to the initial and equilibrium concentration, respectively. The mass (w) of the adsorbent is expressed in grams (g), and the volume (v) of the solution is expressed in liters (L). 

The equilibrium total cyanide adsorption was assessed based on the Langmuir and Freundlich isotherm models. The expression of the Langmuir isotherm model can be represented using the following equation [43]:

(2) $$\frac{C_{e}}{q_{e}}=\frac{1}{K_{L}q_{max}}+\frac{C_{e}}{q_{max}}$$

where q_max_ is expressed in mg g^−1^ and corresponds to the maximum monolayer adsorption, K_L_ is expressed in L mg^−1^ and corresponds to the equilibrium Langmuir constant related to the adsorption energy, and C_e_ is expressed in mg L^−1^ and corresponds to the concentration of solute at equilibrium. Additionally, the R_L_ separation factor values, which offer an idea of the adsorption characteristics, can be represented using the following equation [43]:

(3) $$R_{L}=\frac{1}{\left(1+K_{L}C_{e}\right)}$$

The suitability of the adsorption treatment based on the R_L_ value is given as follows: 0 < R_L_ < 1; it denotes suitable adsorption, R_L_ > 1 unsuitable adsorption, R_L_ = 0 irreversible adsorption, and R_L_ = 1 linear adsorption [43].

Moreover, the Freundlich isotherm model can be represented by the following equation [43]:

(4) qe=KFCe1n

where K_F_ is expressed in L mg^−1^ and corresponds to the Freundlich constant, which specifies the adsorption affinity of the adsorbents, and 1/n is another constant that corresponds to the adsorption intensity. For favorable adsorption, the value of the constant n should be in the range of 1 to 10 [44].

For the thermodynamic studies, the experimental data were fitted according to the parameters of the thermodynamic laws described by Gibbs free energy (∆G^0^, kJ mol^−1^), enthalpy (∆H^0^, kJ mol^−1^), and entropy (∆S^0^, kJ mol^−1^ K^−1^) conventionally used, represented by the following equation [45]

(5) $$∆G^{0}=-RTln\;k_{C}$$

The relationship between ∆G^0^, ∆H^0^, and ∆S^0^, is obtained by the well-known van ’t Hoff equation [45]

(6) $$\ln k_{C}=\frac{-∆H^{0}}{R}\times \frac{1}{T}+\frac{∆S^{0}}{R}$$

where k_L_ (L mg^−1^) is the Langmuir constant and could be achieved as a dimensionless parameter. T is the absolute temperature (K), and R is the universal gas constant (8.314 J mol^−1^ K^−1^). k_C_ is achieved as a dimensionless parameter by multiplying k_L_ by a molecular weight of an adsorbate (M_w_, g mol^−1^) and then by factors 1000 and 55.5, which is the number of moles of pure water contained in a liter, described by the following equation [46].

(7) $$k_{C}=k_{L}\times M_{w}\times 1000\times 55.5$$

The absorption kinetics were estimated by applying pseudo-first-order and pseudo-second-order models, as well as intraparticle diffusion, external-film diffusion, and internal-pore diffusion models. The pseudo-first-order kinetic model is represented by the following equation [44]:

(8) $$\ln\left(q_{e}-q_{t}\right)=\ln\left(q_{e}\right)-k_{1}t$$

where k_1_ is the rate constant (min^−1^) and q_e_ and q_t_ are the cyanide adsorbed per unit weight (mg g^−1^) at equilibrium and at any time t, respectively.

The pseudo-second-order kinetic is represented by the following equation [44]:

(9) $$\frac{t}{q_{t}}=\frac{1}{k_{2}q_{e}^{2}}+\frac{1}{q_{e}}t$$

where k_2_ is the pseudo-second-order rate constant (g mg^−1^ min^−1^).

Finally, to obtain an appropriate understanding of the cyanide adsorption mechanism on the nanoparticles surface, the rate-limiting step in the adsorption process was also estimated. The intraparticle diffusion model supposes that intraparticle diffusion is generally the rate-controlling step in well-mixed solutions. The intraparticle-diffusion model is represented by the following equation [44]:

(10) qt=k3t12+A

where k_3_ (mg g^−1^ min^−1/2^) is the intraparticle diffusion rate constant and A (mg g^−1^) is a constant indicating the width of the boundary layer; that is, the larger the value of A, the greater the boundary layer effect. When the q_t_ plot against the square root of time shows multilinearity, it means that the diffusion occurs in several steps during the process.

The internal pore diffusion model was also utilized in this study to explain the kinetic adsorption data. When the adsorption rate is controlled by particle diffusion, the adsorption rate is represented using the following equation [44]:

(11) $$-ln\left(1-{\left(\frac{q_{t}}{q_{e}}\right)}^{2}\right)=\frac{2\pi^{2}D_{p}}{r^{2}}t$$

On the other hand, regarding external-film-diffusion controls, the adsorption rate is expressed by the following equation [44]:

(12) $$-ln\left(1-\left(\frac{q_{t}}{q_{e}}\right)\right)=\frac{D_{f}C_{s}}{h\;r\;C_{z}}t$$

where q_e_ and q_t_ represent the amount of solute that is taken up by the adsorbent phase at equilibrium and at a specific time t (mg g^−1^), respectively. The ion concentration in the solution and adsorbent are donated by C_s_ (mg L^−1^) and C_z_ (mg kg^−1^), respectively. The variable t refers to the contact time (min), while r represents the average radius of the adsorbent particles (1 × 10^−7^ m). The film thickness around the adsorbent particles is denoted by h and is assumed to be 10^−6^ m in poorly stirred solutions. Finally, the diffusion coefficient in the adsorbent phase is referred to as D_p_ (m^2^ min^−1^), while D_f_ (m^2^ min^−1^) represents the diffusion in the film phase surrounding the adsorbent particles.

### 2.5. Photodegradation Studies

Heterogeneous photocatalysis experiments were performed using the methodology described in our previous study [36]. These experiments were performed in an air-cooled solar box. The batch method was used, keeping the pH of the solution at 7.0 ± 0.1 by adding 0.1 M solutions of hydrochloric acid or sodium hydroxide. Typically, 0.2 g L^−1^ of nanoparticles were magnetically stirred in 500 mL of water containing 20 mg L^−1^ of KCN [38]. The photodegradation rate of cyanide species in the heterogeneous photocatalytic systems exposed to simulated solar light for the TO, La/TO, Ce/TO, and Eu/TO nanoparticles was followed by the Langmuir–Hinshelwood equation [47], which can be represented by the following equation [48]:

(13) $$ln\frac{C_{o}}{C_{t}}=k\;K\;t=k_{app}t$$

where k (min^−1^) is the actual rate constant, K is the adsorption constant of the substrate on the nanoparticles, C_0_ (mg L^−1^) represents the initial concentration of the substrate, and C_t_ (mg L^−1^) represents the concentration at a specific time t (min), and k_app_ (min^−1^) is the apparent rate constant. Plotting ln(C_0_/C_t_) against time t provides the apparent rate constant (k_app_) for substrate degradation from the slope of the curve fit line, and the intercept is equal to zero.

To determine the influence of several reactive oxygen species (ROS) on the reaction system, the radical quenching experiment was performed using isopropanol (i-POH), ethanol (EtOH), and p-benzoquinone (p-BQ). These radical quenchers inhibit h^+^, ^•^OH, and ^•^O_2_^−^, respectively. The experiments were carried out following the methodology described for the heterogeneous photocatalysis experiments but each time incorporating the radical quenchers (0.5 mM) in the reaction system [49]. 

### 2.6. Reuse of Nanoparticles

Finally, in order to verify the reusability of the nanoparticles in cyanide photodegradation, a recycling experiment was designed using the methodology described in our previous study [36]. The recycling experiment was performed for five consecutive cycles. At the end of each treatment cycle, the suspensions were precipitated by leaving them to stand for at least 1 h. After this time, the supernatant liquid was removed and the nanoparticles were carefully washed three times with HPLC water, avoiding loss of solid material. In each treatment cycle, 100 mL of fresh KCN solution (20 mg L^−1^) was used. The amount of nanoparticles used in this experiment was 0.2 g L^−1^ [38].

## 3. Results

### 3.1. Characterization of the Nanoparticles

#### 3.1.1. XRD and SSA Analysis

**Figure 1:**
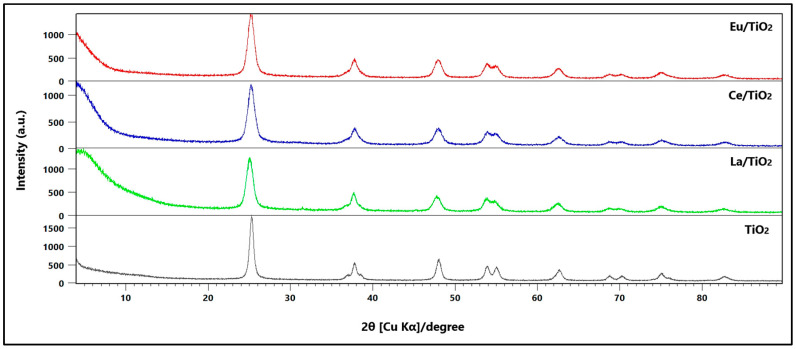
Comparison of XRD patterns of TiO_2_, La/TiO_2_, Ce/TiO_2_, and Eu/TiO_2_ nanoparticles.

Figure 1 shows the diffraction patterns of pristine TiO_2_ compared to TiO_2_ doped with lanthanum (La/TO), cerium (Ce/TO), and europium (Eu/TO). These nanoparticles were synthesized at 500 °C. The diffraction peaks of anatase phase (TiO_2_) at 2θ values of 25.30°, 36.95°, 37.79°, 38.57°, 48.04°, 53.89°, 55.06°, 62.11°, 62.69°, 68.76°, 70.29°, 75.04°, 76.03°, 82.67°, and 83.15° were assigned to planes (1 0 1), (1 0 3), (0 0 4), (1 1 2), (2 0 0), (1 0 5), (2 1 1), (2 1 3), (2 0 4), (1 1 6), (2 2 0), (2 1 5), (3 0 1), (2 2 4), and (3 1 2), respectively. The TiO_2_ crystal structure was indexed to tetragonal phase with unit cell parameters a = b = 3.79 Å and c = 9.51 Å, α = β = γ = 90°, unit cell volume = 136.30 Å^3^, and space group |41/amd(141) according to standard card JCPDS card No. 01-073-1764. Due to the low concentration of the doping elements, the maximum diffraction peaks reported in the literature are not observed for La_2_O_3_ (2θ = 29.96°) [50], Ce_2_O_3_ (2θ = 30.39°) [51], CeO_2_ (2θ = 28.57°) [52], nor for europium oxide (2θ = 28.44°) [53]. 

The crystal sizes of the TO, La/TO, Ce/TO, and Eu/TO nanoparticles were calculated considering the most intense diffraction peak. The Scherrer equation (Equation (14)) was used for the respective calculation [54]

(14) $$A=\frac{K\lambda }{\beta \;cos\theta }$$

where A is the size of the crystals expressed in nm, λ = 0.15406 nm and K = 0.89 represent the wavelength of the X-ray beam and the shape factor, respectively, θ represents the Bragg angle, while β represents the full width at half peak height maximum (FWHM) of the X-ray diffraction peak. The average crystal sizes of the TO, La/TO, Ce/TO, and Eu/TO nanomaterials were calculated at 28.54 (±0.98), 19.24 (±0.65), 20.68 (±0.87), and 21.94 (±1.07) nm, respectively.

On the other hand, the specific surface area (SSA) was evaluated by the single-point BET (Brunauer–Emmet–Teller) method. The SSA of the pristine TiO_2_ (TO) was measured as 88 m^2^ g^−1^, which was slightly lower than that of the La/TO, Ce/TO, and Eu/TO nanoparticles, with values of 126, 104, and 96 m^2^ g^−1^, respectively. From these results, it is suggested that elements lanthanum (La), cerium (Ce), and europium (Eu) have a specific effect on inhibition of growth of TiO_2_ crystallites, stabilization of this oxide in its anatase phase, as well as increasing specific surface area (SSA).

#### 3.1.2. FTIR Analysis

**Figure 2:**
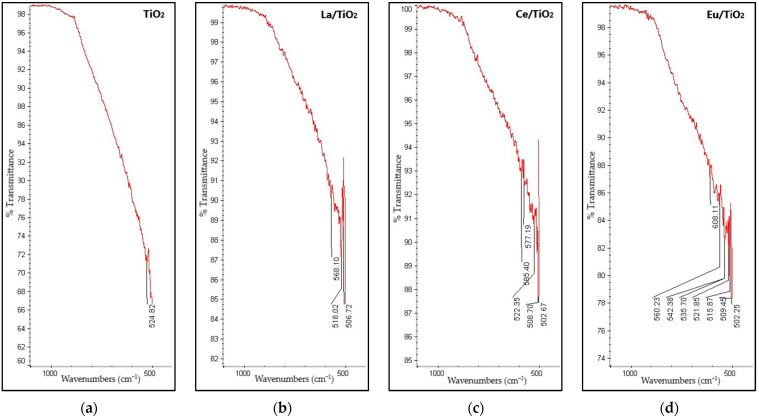
FTIR spectra of (**a**) TiO_2_, (**b**) La/TiO_2_, (**c**) Ce/TiO_2_, and (**d**) Eu/TiO_2_ nanoparticles.

FTIR spectroscopy was used for chemical characterization of TO, La/TO, Ce/TO, and Eu/TO nanoparticles. The FTIR spectra of all the synthesized nanoparticles are shown in Figure 2a–d, respectively.

The FTIR spectrum of TiO_2_ reveals the existence of a broad band in the region of 540–900 cm^−1^, which is assigned to Ti–O and Ti–O–Ti stretching vibrations [55,56]. After doping, the main change was identified in this region, presenting new unresolved bands around the 500–600 cm^−1^ region that could be attributed to Ln–O stretching modes of vibrations. The results obtained in this analysis agree with those reported in the literature [57,58].

On the other hand, the spectra in Figure 2 show that there is a tendency to increase the vibrational frequencies as the ionic radius of the doping elements decreases. Evidence from the literature indicates that Ln–O single-bond stretching frequencies and Ln–O single-bond stretching force constants show a linear correlation with inverse ionic radii [59].

#### 3.1.3. SEM and EDS Analysis

**Figure 3:**
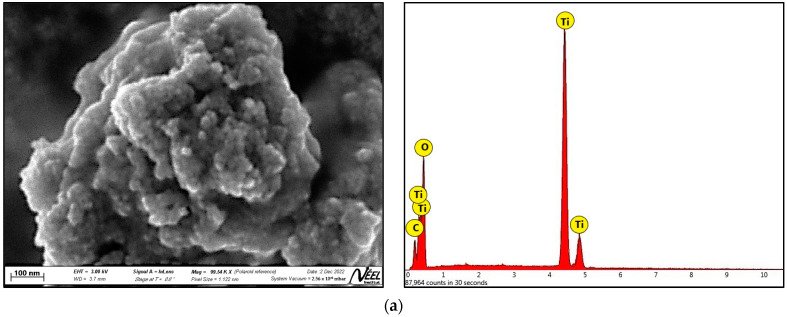

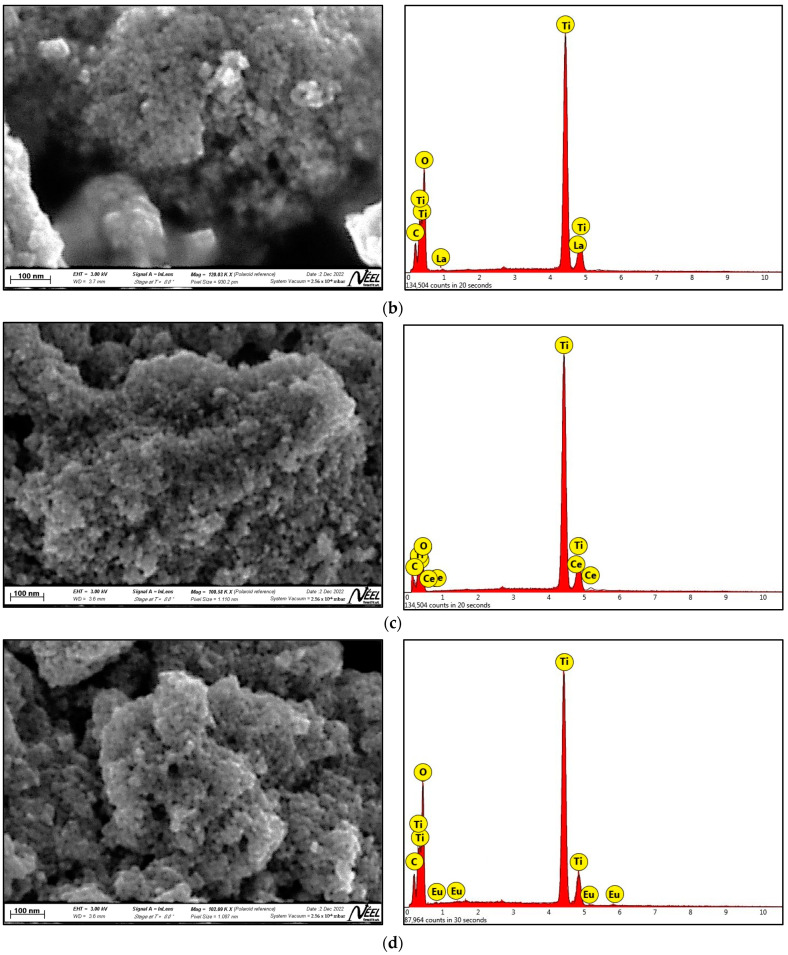
SEM images and EDS spectra of (**a**) TO, (**b**) La/TO, (**c**) Ce/TO, and (**d**) Eu/TO nanoparticles.

Figure 3a–d displays the SEM photomicrographs of TO, La/TO, Ce/TO, and Eu/TO nanoparticles, respectively. In these figures, it is evident that all the nanoparticles appear almost spherical and are highly agglomerated. These results are in agreement with those informed by other authors [60]. Doped nanoparticles are smaller than pristine TO nanoparticles. The average size of the TO nanoparticles was 32 nm, in contrast to La/TO, Ce/TO, and Eu/TO nanoparticles, which had mean sizes of 26, 27, and 28 nm, respectively. The size of the synthesized nanoparticles was measured using ImageJ2, which is public domain software for scientific image processing and analysis [61,62]. The sizes of the nanoparticles are within the range of values reported by other authors [63]. 

The SEM images presented in this study support the results of the XRD analysis (Figure 1), demonstrating that doping elements lanthanum, cerium, and europium are effective in preventing crystallite growth and stabilizing the structure of the TiO_2_ semiconductor.

Likewise, Figure 3a–d displays the EDS spectra of TO, La/TO, Ce/TO, and Eu/TO nanoparticles, respectively. These spectra support the results of the FTIR analysis (Figure 2), thus confirming incorporation of lanthanide elements La (1.32 wt%), Ce (1.34 wt%), and Eu (1.28 wt%) on the TiO_2_ structure. 

#### 3.1.4. Optical and Photoelectric Properties

**Figure 4:**
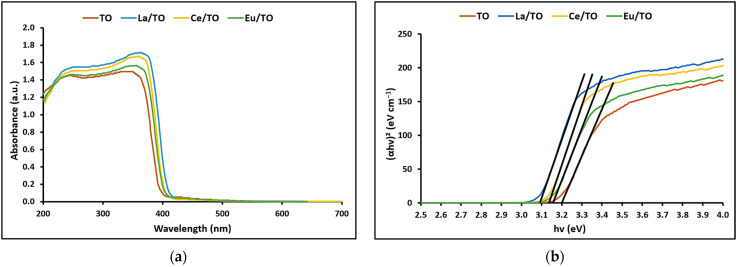
(**a**) UV–vis diffuse reflectance spectra and (**b**) plots of (αhv)^2^ vs. hv of TO, La/TO, Ce/TO, and Eu/TO nanoparticles.

The optical absorption capacity of the synthesized nanoparticles was determined by UV–visible (UV–vis) DRS at room temperature in the range of 200 to 700 nm. Figure 4a shows the DRS UV–vis spectrum of TO, La/TO, Ce/TO, and Eu/TO nanoparticles. Comparatively, the absorption behavior in the visible light spectrum (around 400 nm) was slightly enhanced for La/TO, Ce/TO, and Eu/TO. Due to this bathochromic shift, it is suggested that, compared to TO, doped nanoparticles have improved response to visible light.

The Tauc formula was used to estimate the direct bandgap energy (E_g_) for the nanoparticles using the following equation [64]:

(15) αhv=Ahv−Egn2

where α is the absorption coefficient, hv is photon energy, computed by hv = 1240/λ, A is a constant that is correlated with the absorption brink width parameter, and n is the optical transition phase. The value of n depends on the semiconductor transitions, that is, n = 1 for direct transitions and n = 4 for indirect transitions [64].

The relationship between (αhv)^2^ and photon energy (hv) shown in Figure 4b enabled estimating the direct bandgap energy (E_g_) [65]. Based on the data presented in this figure, the direct E_g_ values achieved from the intersections of the straight line with the energy axis [66] were 3.20, 3.10, 3.14, and 3.16 eV for TO, La/TO, Ce/TO, and Eu/TO, respectively.

### 3.2. Effect of Nanoparticles Composition

**Figure 5:**
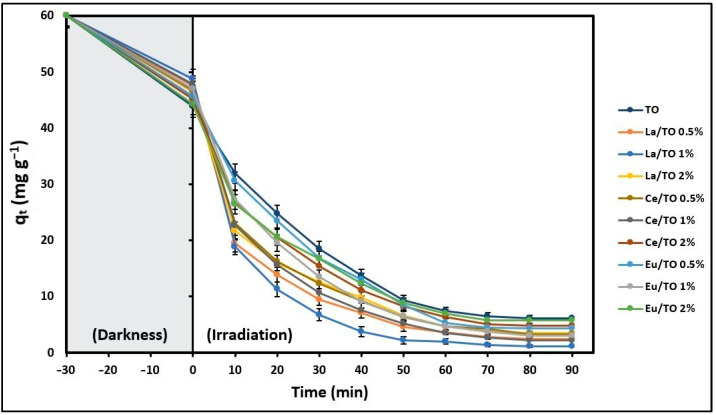
Cyanide removal capacity as a function of nanoparticles composition (catalyst concentration = 0.2 g L^−1^; cyanide concentration = 20 mg L^−1^; solution pH = 7.0 ± 0.1).

The effect of the composition of TO, La/TO, Ce/TO, and Eu/TO nanoparticles on their total cyanide removal capacity q_e_ (mg g^−1^) was evaluated in the absence of light (30 min) and then under simulated solar light. The results of this experiment are shown in Figure 5. For all the nanoparticles, it was found that, after 90 min of radiation exposure and under the test conditions, it is possible to achieve maximum removal. This time was denoted as the equilibrium time t_e_ (min).

The findings of the analysis of variance (ANOVA) carried out at t_e_ (min) are summarized in Table 1. The values in the same column that have been assigned different letters (a–g) are significantly different from each other, with a high level of statistical significance (*p* < 0.01). Based on these results, it is suggested that, for each dopant, the best composition was 1.0 wt.%; therefore, the La/TO (1%), Ce/TO (1%), and Eu/TO (1%) nanoparticles were selected to perform cyanide removal tests and evaluate the effectiveness of doping in relation to non-doped TiO_2_.

### 3.3. Adsorption Studies

#### 3.3.1. Effect of pH on Cyanide Adsorption

**Figure 6:**
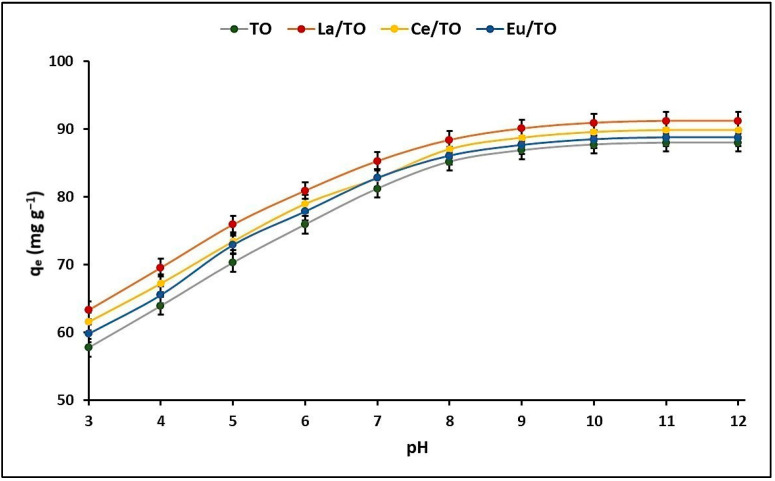
Solution pH effect experiment.

Figure 6 shows the results of the cyanide adsorption experiment as a function of the pH of the solutions. Total cyanide adsorption capacity (HCN + CN^−^) remained unchanged in the pH range 9 to 12 for pristine TO (pH_PZC_ = 6.9) and for doped nanoparticles (pH_PZC_ = 7.1). The point of zero charge (PZC) corresponds to the pH value at which the net surface charge of the solid is equal to zero under certain conditions of pressure, temperature, and composition of the aqueous solution [67]. The point of zero charge (PZC) for all nanoparticles was determined at room temperature (21 ± 2 °C) using the pH drift procedure (ΔpH = pH_f_ − pH_i_ = 0). The experiment was carried out in 50 mL tubes, in which 25 mL of a 0.1 M NaCl solution and 0.1 g of solid sample were placed. The pH values of the solutions contained in the tubes were adjusted to values between 3 and 12 using 0.1 M solutions of HCl or NaOH. These initial pH values were designated as pH_i_. The tubes were shaken for 24 h at 230 rpm. After this time, the final pH of the supernatant liquid in each tube was measured and designated as pH_f_. The PZC was determined from the graph of ΔpH (ΔpH = pH_f_ – pH_i_) vs. pH_i_. The procedure was repeated for all nanoparticle samples using 0.01 and 0.05 M NaCl solutions. All experiments were carried out in triplicate, and, for each sample, the average pH_PZC_ value was reported [68].

In this study, the pH_PZC_ of all nanoparticles was on average 7.0. This implies that, at pH > pH_PZC_ = 7.0, the surface of the nanoparticles has a negative charge and, at pH < pH_PZC_ = 7.0, it has a positive charge.

The ability of ions to be adsorbed on a surface from a solution is greatly influenced by the pH of the solution. This is because pH affects both the surface charge of the ion adsorbent and the extent to which the ions ionize and exist in different forms. Therefore, to better understand the adsorption mechanisms of cyanide species on the surface of nanoparticles, it is important to consider the pH-dependent distribution of cyanide species in solution (Equation (16)) [7].

(16) $$HCN↔{CN}^{-}+H^{+}\;\;\;\;\;\;pKa\;=\;9.4$$

Inorganic cyanides, such as KCN and NaCN, are weak acids and hydrolyze to form hydrocyanic acid (HCN), which has a pKa value of 9.4. At pH < 9.4, HCN is the predominant species in the aqueous solution, while, at pH > 9.4, HCN dissociates into H^+^ and CN^−^ ions [69]. CN^−^ ions have nucleophilic characteristics and experience a strong electrostatic attraction in solutions at pH > 9.0, which is why several authors have informed higher cyanide adsorption at pH values between 9 and 11 [42]. However, other authors have also informed that inorganic cyanides can easily dissociate to form HCN and CN^−^ species at a neutral pH of 7.0 [70]. In fact, several investigations related to cyanide adsorption have been carried out at pH values around 7.0 (pH < pKa), suggesting that the high adsorption of HCN species is based on interactions with the existing Lewis acids sites on the adsorbent surface [71,72]. Consequently, the adsorption experiments were performed at a pH of 7.0.

#### 3.3.2. Maximum Cyanide Adsorption Capacity

**Figure 7:**
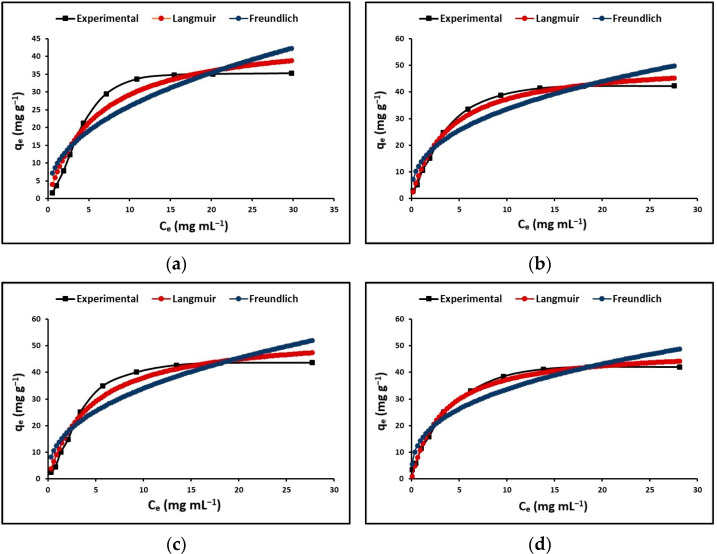
Adsorption isotherms of (**a**) TO, (**b**) La/TO, (**c**) Ce/TO, and (**d**) Eu/TO.

In this study, the Langmuir and Freundlich isotherms were examined as equilibrium models that rely on the initial concentration. The Langmuir isotherm represents a credible theoretical framework for the process of adsorption occurring on a uniform and completely homogeneous surface, whereby a finite quantity of identical and specific sites are available for adsorption. In this model, there is minimal interaction among the molecules. The Freundlich equation is a non-theoretical formula that does not assume uniformity in the energy of surface sites, allows for unlimited adsorption capacity, and corresponds to an exponential distribution of active sites that reflects a heterogeneous surface. Figure 7 shows the cyanide adsorption isotherms of TO, La/TO, Ce/TO, and Eu/TO nanoparticles. This figure clearly shows that, for all nanoparticles, the Langmuir model is a more suitable description of the adsorption process compared to the Freundlich model.

The values estimated at different temperatures for the constants of the Langmuir and Freundlich models are given in Table 2. In this table, it is evident that the values of the R_L_ separation factor or equilibrium parameter were in the range of 0 to 1, while the values of the coefficient n, which represents the intensity of adsorption, were in the range of 1 to 10. Consequently, it is suggested that adsorption of cyanide species on the surface of all materials was satisfactory.

#### 3.3.3. Adsorption Thermodynamics

**Figure 8:**
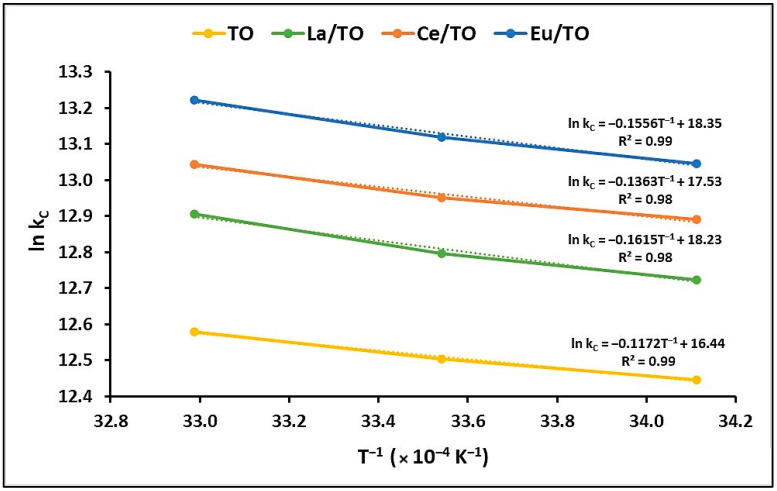
Thermodynamic study of cyanide adsorption onto nanoparticles.

The thermodynamic parameters provide information about the spontaneity and possibility of a process. To determine these parameters, namely Gibbs free energy change (∆G°), enthalpy change (∆H°), and surface entropy change (∆S°), the equilibrium constant was measured at different temperatures, as shown in Figure 8. 

The results of the thermodynamic parameters are shown in Table 3. ∆G° indicates the degree of spontaneity of the process; negative values reflect higher adsorption favorability. Likewise, negative ΔH° values reflect that the process is exothermic, while negative ΔS° values indicate a decrease in randomness at the solution–solid interface during adsorption.

#### 3.3.4. Kinetic Behavior of Cyanide Adsorption

**Figure 9:**
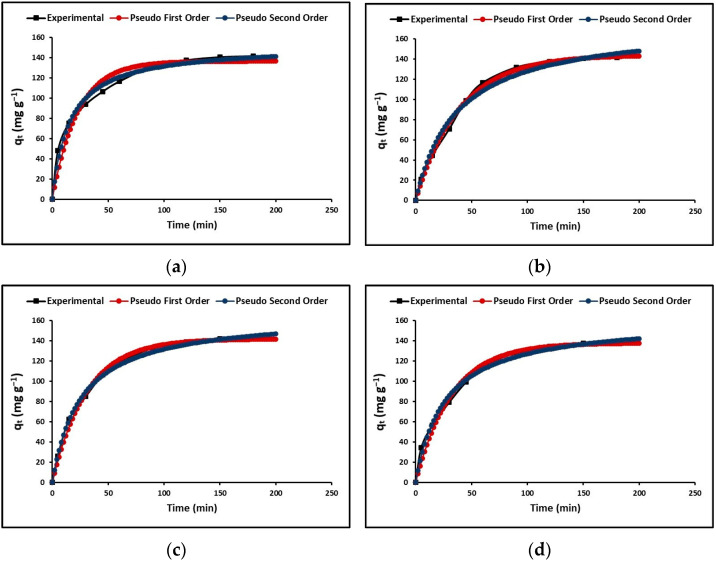
Adsorption kinetics of (**a**) TO, (**b**) La/TO, (**c**) Ce/TO, and (**d**) Eu/TO.

To design and evaluate adsorbents for adsorption, it is important to determine the rate of the time-dependent process. Two models, the pseudo-first-order (Lagergren) and pseudo-second-order (Ho) models, were used to describe the adsorption kinetics, which are commonly used as simplified models. Both models, as shown in Figure 9, exhibit a rapid initial adsorption stage followed by a plateau stage. Table 4 indicates that the correlation coefficient of the pseudo-second-order model is higher than that of the pseudo-first-order model, suggesting a chemisorption process, according to previous literature [9].

**Figure 10:**
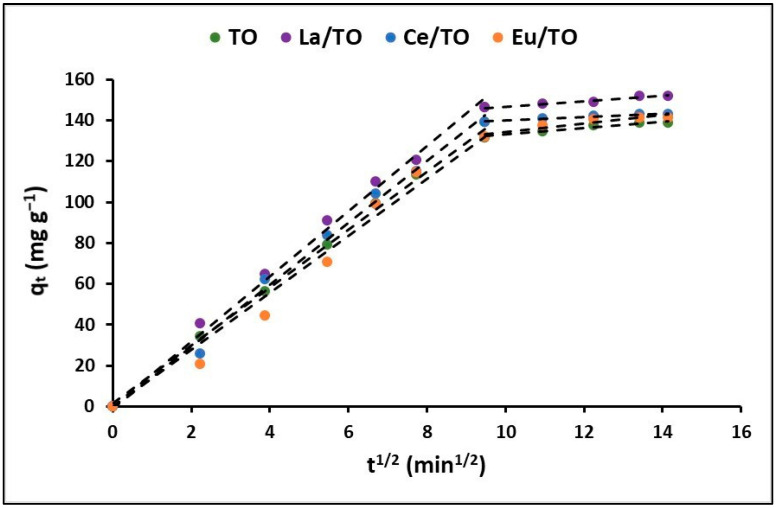
Intraparticle diffusion plots for cyanide removal by nanoparticles.

On the other hand, the intraparticle diffusion model was utilized to explain the adsorption rate, which is dependent on the rate at which the cyanide species transfer from the aqueous solution to the adsorption sites on the nanoparticles. Figure 10 shows the variation in the q_t_ (mg g^−1^) curves as a function of time (t^1/2^) for the TO, La/TO, Ce/TO, and Eu/ZTO nanoparticles.

Table 4 shows the total cyanide adsorption kinetic parameters estimated in this study for all nanoparticles.

### 3.4. Photodegradation Studies

#### 3.4.1. Kinetics of Cyanide Photodegradation

TiO_2_ is a widely used photocatalyst to efficiently degrade organic compounds due to its strong oxidizing capacity, which is generated when subjected to the action of light. In this study, the photoactivity of pristine TiO_2_ nanoparticles and La/TiO_2_, Ce/TiO_2_, and Eu/TiO_2_ nanoparticles was tested through model cyanide photodegradation reaction in aqueous solutions under simulated solar radiation.

**Figure 11:**
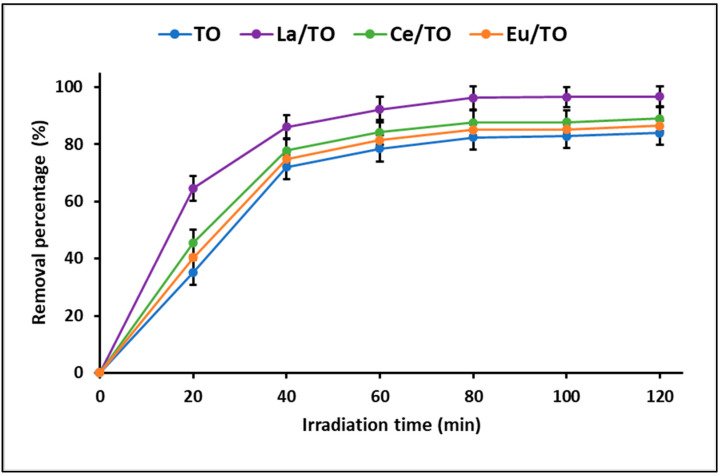
Photocatalytic cyanide degradation by nanoparticles.

The Langmuir–Hinshelwood equation showed a linear correlation between ln(C_0_/C_t_) and t, confirming that the photocatalytic degradation reaction proceeds via a pseudo-first-order reaction. The calculated apparent rate constants (k_app_) were calculated to be 0.016, 0.030, 0.018, and 0.017 min^−1^ for TO, La/TO, Ce/TO, and Eu/TO nanoparticles, respectively. These results are in agreement with those reported by other authors [48,73]. The results obtained in the photocatalytic degradation test are shown in Figure 11. From this figure, the maximum percentage of cyanide degradation for all nanoparticles is reached around the first 90 min, after which photodegradation becomes almost constant. Evidence from the literature suggests that there is a limit to the efficacy of photocatalysis for complete degradation of some pollutants [74]. In this study, maximum efficiency was reached by La/TO (96.8%), followed by Ce/TO (88.9%), Eu/TO (86.5%), and TO (83.9%).

#### 3.4.2. Effect of the Photogenerated Radicals

**Figure 12:**
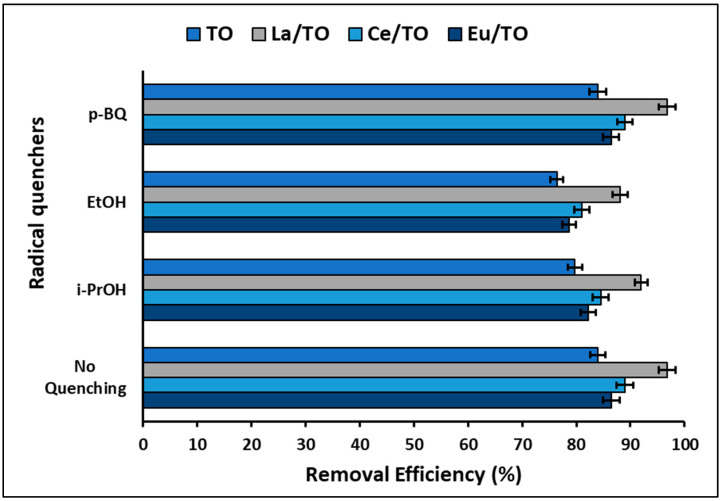
Photogenerated radical quenching experiments.

It is widely known that photogenerated electrons (e^−^), photogenerated holes (h+), hydroxyl radicals (^•^OH), and superoxide radicals (^•^O_2_^−^) are the main reactive oxygen species (ROS) in photocatalytic processes. Therefore, to establish the impact of various ROS on the reaction system, a radical quenching experiment was performed incorporating different radical quenchers [75]. Figure 12 shows the results of this experiment. From this figure, it is evident that, for all nanoparticles, the efficiency of photocatalytic degradation of total cyanide decreased with introduction of isopropanol (i-PrOH) and ethanol (EtOH), which are quenchers of h^+^ and ^•^OH radicals, respectively. On average, the nanoparticles decreased their cyanide removal efficiency by 9.0% and 5.0% when introducing ethanol and isopropanol, respectively. On the other hand, introduction of p-Benzoquinone (p-BQ) as a quencher of radials ^•^O_2_^−^ did not affect the efficiency of the nanoparticles for cyanide removal.

### 3.5. Total Efficiency and Reuse of Nanoparticles

**Figure 13:**
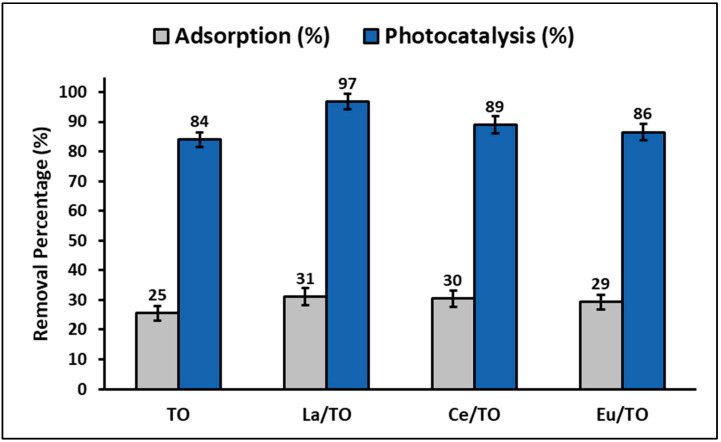
Percentage of cyanide adsorbed and photodegraded by nanoparticles.

The percentage of cyanide adsorbed and photodegraded by the synthesized nanoparticles is shown comparatively in Figure 13. In this figure, for all the nanoparticles, the photocatalysis process was more efficient for removal of cyanide species than the adsorption process. From this figure, it can be inferred that, under the conditions tested in this study, the highest adsorption and photodegradation capacity of cyanide species was achieved by La/TO nanoparticles, followed by Ce/TO, Eu/TO, and finally by TO nanoparticles.

**Figure 14:**
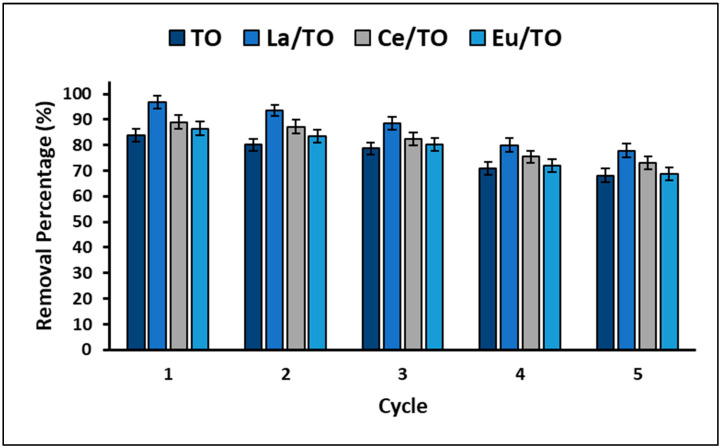
Nanoparticle reuse experiment.

Finally, knowing that the stability and recyclability of materials with adsorbent and photocatalytic applications are considered crucial aspects for their large-scale application, in this study, reuse experiments were performed for five consecutive cyanide removal cycles. Figure 14 shows the removal efficiency of the synthesized nanoparticles during the five cycles.

## 4. Discussion

### 4.1. Characterization of Nanoparticles

#### 4.1.1. XRD and SSA Analysis

The results depicted in Figure 1 indicate that doping with lanthanide (Ln) can impact the crystal structure of pure TiO_2_ (anatase), causing slight broadening and reduction in intensity of its characteristic diffraction peaks. This suggests that the adsorbent and photocatalytic properties of this semiconductor oxide can be affected by the presence of Ln dopants [76]. According to the literature, it is very difficult for lanthanide ions to substitute Ti^4+^ ions in the TiO_2_ crystal lattice. This is primarily due to the significant difference in ionic radii between Ti^4+^ (0.068 nm) and the lanthanide ions, such as La^3+^ (0.116 nm), Ce^3+^ (0.114 nm), Ce^4+^ (0.101 nm), and Eu^3+^ (0.107 nm). Therefore, La^3+^, Ce^3+/4+^, and Eu^3+^ could rather be uniformly dispersed on the TiO_2_ surface in the form of La_2_O_3_, Ce_2_O_3_/CeO_2_, and Eu_2_O_3_ particles, respectively [77]. It should be noted that, even though the doping elements were present in low concentration (1 wt%) and were evenly dispersed on the TiO_2_ surface, Figure 1 does not exhibit identifiable diffraction peaks of the corresponding lanthanide oxides [78]. Nevertheless, the reduction in crystallite size may be attributed to the existence of La–O–Ti, Ce–O–Ti, or Eu–O–Ti bonds on the surface of TiO_2_. These findings are consistent with those reported by other researchers, who have demonstrated a decrease in crystallite size caused by formation of Ln–O–Ti bonds [79].

The results of the BET analysis would corroborate this appreciation since the doped nanoparticles (La/TO, Ce/TO, and Eu/TO) show higher specific surface area related to pristine TiO_2_. From the results of the BET analysis, it is suggested that La/TO, Ce/TO, and Eu/TO nanoparticles could have better cyanide species removal capacity than TO due to the greater accessibility of active sites on the increased surface area. Furthermore, the high surface area of the doped nanoparticles could facilitate diffusion of cyanide species and photogenerated ROS. In this way, photodegradation of cyanide could be improved by allowing more energetic photons to be adsorbed on the surface of the photocatalytic nanoparticles. Finally, the high surface area also facilitates contact between the nanoparticles and the cyanide species present in the aqueous medium, which benefits the subsequent photocatalytic reaction [48].

#### 4.1.2. FTIR Analysis

The FTIR spectra shown in Figure 2b–d allowed to demonstrate that, due to doping, the broad band of the TiO_2_ semiconductor, shown in the region of 540–900 cm^−1^ in Figure 2a, changes drastically. According to the literature, this change can be attributed to the presence of Ln-–O stretching modes of vibrations [24]. Unresolved bands suggest the existence of uniform stresses in the lattice, which is generally associated with defects in the material structure. These defects, probably derived from doping, can cause shortening of the Ti–O bond, promoting vibrational mode changes observed in doped nanoparticles relative to pristine TiO_2_ [80]. The results obtained in this analysis agree with those reported in the literature [57,58].

#### 4.1.3. SEM and EDS Analysis

Figure 3 shows a comparison of the SEM micrographs of the TO, La/TO, Ce/TO, and Eu/TO nanoparticles. As evidenced in this figure, lanthanide elements La, Ce, and Eu, by acting as TiO_2_ dopants, could promote reduction in size of semiconductor crystallites. This is probably due to the fact that dispersion of dopant ions La^3+^, Ce^3+/4+^, and Eu^3+^ on the TiO_2_ surface could restrict direct contact between neighboring crystallites of this semiconductor, thus inhibiting their growth [34]. According to the literature, restriction of direct contact between neighboring crystallites is due to generation of large defects in the crystal lattice of the material. These defects can change the interatomic distance and, therefore, affect the stability of the bonds between TiO_2_ atoms. As a result, TiO_2_ microcrystals with different sizes and random orientations can be formed [81]. The results of this study agree with those reported by other authors, who have shown that doping enables stabilization of small particles [76]. Regarding EDS analysis, the results obtained allowed us to confirm the presence of La (1.32 wt%), Ce (1.34 wt%), and Eu (1.28 wt%) on the TiO_2_ structure. These results support the results obtained in the XRD and FTIR assays. 

#### 4.1.4. Optical and Photoelectric Properties

Figure 4a displays the UV–vis absorption spectra of TO, La/TO, Ce/TO, and Eu/TO nanoparticles. In this study, the absorption threshold of the pristine TiO_2_ (TO) semiconductor was around 350 nm. The strong absorption band observed in this region for TO would be associated with electronic transition from the valence band (VB) dominated by the 2p orbital of O to the conduction band (CB) dominated by the 3d orbital of Ti [35]. In contrast to the TO spectrum, the spectra of the nanoparticles that have been doped exhibit a red shift. This phenomenon has been described in the literature as being caused by a charge transfer transition between the f-electrons of the lanthanide ions and either the conduction band or valence band of TiO_2_ [82]. As can be seen in Figure 4a, the shift of the absorption edge towards a slightly longer wavelength depends on the lanthanide ions used as dopants; however, it is evident that doping with these ions could improve the response of TO to visible light.

On the other hand, to estimate the bandgap energy (E_g_) of the synthesized nanoparticles, the (UV–vis) DRS method was used, and the absorption data were fitted to equations for direct bandgap transitions. Figure 4b shows a reduction in the E_g_ value for the doped nanoparticles relative to the value calculated for the pristine TiO_2_. According to the literature, the reduction in the value of E_g_ is due to formation of impure energy levels (impure orbitals) between the VB and the CB of the doped semiconductor [83]. Furthermore, due to the presence of these impure orbitals, less energy is required to achieve the transfer of electrons from the valence band to the impure orbital and/or from the latter to the conduction band. From these results, it is suggested that doping of the TiO_2_ semiconductor with lanthanide elements La, Ce, and Eu allows to reduce the bandgap energy and, consequently, to optimize the capacity of TiO_2_ to absorb wavelengths with less energy (including solar radiation), as well as reduce recombination of photogenerated charges and modify the adsorption capacity of the surface of this semiconductor [13].

### 4.2. Effect of Nanoparticles Composition

In this study, a preliminary experiment was developed in order to evaluate the best concentration of elements La, Ce, and Eu as TiO_2_ dopants for effective removal of total cyanide from aqueous systems. Figure 5 shows that the total cyanide removal capacity of the doped nanoparticles increases when the percentage by weight of the dopant in the semiconductor increases from 0.5 to 1%. However, the total cyanide removal capacity of the doped nanoparticles decreases when the percentage by weight of the dopant in the semiconductor increases from 1 to 2%. According to the literature, the reason for this is that the presence of high concentrations of doping elements can lead to an increase in the number of oxygen vacancies in the material, resulting in an increase in the number of recombination centers for photoinduced charges [84]. Similarly, when the dopant particles agglomerate and form clusters, they can obstruct the active sites on the surface of the semiconductor, leading to a reduction in its photocatalytic efficiency. This has been reported in the literature as well [85]. These findings align with previous studies conducted by other researchers, who have shown that incorporating lanthanide elements at levels of 1–2 wt% can effectively enhance photoactivity of semiconductors across a broad range of the electromagnetic spectrum [78].

### 4.3. Adsorption Studies

#### 4.3.1. Effect of pH on Cyanide Adsorption

Due to the protonation–deprotonation balance of the surface hydroxyl groups, the surface of titanium oxide (TiO_2_) has a pH-dependent charge, so this oxide presents a positive charge under acidic conditions and a negative charge under basic conditions [67]. In the middle of these two regions is the point of zero charge (PZC), that is, the pH where the total charge on the TiO_2_ surface is zero. As reported in the literature, adsorption of ionic doping species, including lanthanides La, Ce, and Eu, could change the surface charge density and position of PZC of TiO_2_ [86,87].

Several studies have reported that TiO_2_ behaves as a weak Brönsted acid and that, when hydrated, it can form surface hydroxyl groups (Ti–OH) [88]. The hydroxyl groups present can participate in chemical reactions involving association and dissociation of protons, thus generating a pH-dependent surface charge:

(17) $$TiOH+H^{+}↔Ti{OH}_{2}^{+}\;\;\;\;\;\;\;\;\;\;\;\;\;\;\;at\;pH\;<\;{pH}_{PZC}$$

and

(18) $$TiOH↔{TiO}^{-}+H^{+}+H_{2}O\;\;\;\;\;\;\;\;\;\;\;\;at\;pH\;>\;{pH}_{PZC}$$

where the positive, neutral, and negative surface hydroxyl groups are represented by TiOH_2_^+^, TiOH, and TiO^−^, respectively [87]. 

From Figure 6, it can be inferred that, as the pH of the solution enhances, the amount of total cyanide adsorbed also increases until reaching an adsorption maximum at pH = 9. Above this value, cyanide adsorption remains constant for all nanoparticles. In this figure, it is also observed that the doped nanoparticles have a higher total cyanide removal capacity compared to pristine TiO_2_. This is probably because doping with lanthanum (La), cerium (Ce), and europium (Eu) enables greater total number of available active sites on the surface of TiO_2_ nanoparticles, thus driving favorable kinetics [34]. 

According to the literature, the pH level of a solution has an impact on the surface charge of the adsorbents and the ionization and speciation of the adsorbate. Pristine and doped TiO_2_ nanoparticles have a pH_PZC_ of 6.9 and 7.1, respectively. This implies that, at higher pH levels, there is a preference for an increase in negatively charged groups, while, at lower pH levels, there is a preference for an increase in positively charged groups. Regarding cyanide (pKa = 9.4), at pH values < pKa, this molecule in solution associates in its molecular form (HCN), while, at pH > pKa, it dissociates mainly in its ionic form (CN^−^ + H^+^).

Evidence from several studies indicates that, at pH < pH_PZC_, the specific adsorption of HCN species on the OH groups present on the surface of hydrated nanoparticles is driven through formation of N–H polar covalent bonds, as indicated below [69]

(19) TiOH+HCN↔TiOH⋯NCH

or

(20) $$Ti{OH}_{2}^{+}+HCN↔Ti{OH}_{2}^{+}\cdots NCH$$

For pH < 7.0, the surface of the nanoparticles exhibits a positive charge and the degree of HCN dissociation is negligible. Therefore, electrostatic forces of attraction between a charged surface and a neutral molecule are unlikely to develop, especially at very low pH values. At pH > 7.0, the negative charge on the surface of the nanoparticles increases, but the dissociation of HCN into CN^−^ ions is negligible until pH = pKa = 9.4 is reached. However, in the pH range between 7.0 and 9.4, an increase in the forces of electrostatic attraction is expected and, therefore, an increase in the adsorption of HCN species on the negative surface of the nanoparticles. At pH > pKa = 9.4, the proportion of CN^−^ species in the solution increases, but, in parallel, the surface of the nanoparticles becomes more negative since pH > pH_PZC_. This generates an augment in the repulsive forces between the negative surface sites of the nanoparticles and the negative CN^−^ ions, which causes a reduction in the adsorption capacity of these ions at very high pH values, as shown in Figure 6.

The strong dependence between the pH of the solution and the adsorption capacity of the nanoparticles is a clear indication that, in this study, the total cyanide adsorption mechanism was mainly driven by electrostatic interactions, although other types of interactions cannot be excluded (van der Waals and/or specific interactions). In fact, it is suggested that adsorption of CN^−^ species on La/TO, Ce/TO, and Eu/TO nanoparticles could occur predominantly by chemical adsorption instead of physical adsorption (outer sphere complex), although the presence of physical adsorption is not ruled out completely. The results of this study are consistent with those reported in the literature [44]. When CN^−^ is in contact with active cationic sites on the adsorbent surface, it forms a bond with them. According to the literature, lanthanum, cerium, and europium ions show strong electron withdrawal effect [25,33], so the presence of these lanthanides on the surface of TiO_2_ nanoparticles contributes to generation of Lewis acids sites [28,29,89]. It should be mentioned that these active cationic sites could certainly improve cyanide adsorption on the TiO_2_ surface [34], in addition to providing this semiconductor with catalytic stability in an aqueous reaction system [90].

#### 4.3.2. Maximum Cyanide Adsorption Capacity

Figure 7 shows the adsorption isotherms obtained for the synthesized nanoparticles. This figure displays that, at low concentrations of cyanide species (HCN + CN^−^), the adsorption rate increases; instead, at high concentrations of these species, the adsorption rate reaches a maximum where it stabilizes. One possible reason for this phenomenon is the excessive presence of cyanide compounds that compete for the available active sites on the surface of the adsorbent nanoparticles. Evidence from these results suggests that the initial concentration of cyanide species in solution could generate a significant driving force to allow these species to migrate from the liquid phase to the nanoparticle surface [91].

The adsorption data obtained in this study for TO, La/TO, Ce/TO, and Eu/TO nanoparticles were fitted to the Langmuir and Freundlich isotherm models. Table 2 describes the parameters corresponding to adjustment of the experimental data. As shown in this table, given the values of the correlation coefficient (R^2^), it is concluded that the Langmuir model is the most suitable to describe the equilibrium adsorption behavior. Consequently, it is suggested that monolayer adsorption of cyanide species on the synthesized nanoparticles proceeds as a phenomenon of electrostatic attraction. This attraction takes place in regions with homogeneous surfaces, where the strongest binding sites are initially filled, and, as the saturation level increases, the binding strength decreases [44]. Finally, the values of the Langmuir and Freundlich constants (R_L_ and n) shown in Table 2 confirm the favorable adsorption of cyanide species on the nanoparticles synthesized in this study.

#### 4.3.3. Adsorption Thermodynamics

As mentioned above, thermodynamic parameters offer the most reliable indications for effective implementation of a process in practice. Table 3 shows the thermodynamic parameters obtained in this study for removal of cyanide species. Negative ∆G° values suggest the feasibility and thermodynamic spontaneity of total cyanide adsorption on TO, La/TO, Ce/TO, and Eu/TO nanoparticles. Furthermore, the decrease in negative values of ∆G° with increasing temperature reveals an increase in the effectiveness of the adsorption process at high temperatures. On the other hand, the positive values of ∆H° and ∆S° shown in Table 3 suggest that adsorption of cyanide species on the nanoparticles occurred as an endothermic process. Finally, the positive ∆S° also suggests an increase in randomness at the solution/solid interface, with several structural alterations in the active sites of the nanoparticles during cyanide fixation. These results are in agreement with those informed by other authors [43].

#### 4.3.4. Kinetic Behavior of Cyanide Adsorption

The kinetic models of adsorption allow to determine the contact time necessary for the whole adsorption of the chemical species. Figure 9 shows the fit of the experimental data obtained in this investigation with the pseudo-first-order and pseudo-second-order kinetic models. In this figure, for all nanoparticles (TO, La/TO, Ce/TO, and Eu/TO), the concentration of cyanide species decreases very fast at the beginning of the process and tends to be constant after ~90 min. The rapid adsorption that occurs in the initial stage could be due to the high concentration gradient, as well as the presence of vacant adsorption sites. Table 4 shows the kinetic parameters of adsorption obtained for all the nanoparticles. From the values shown in this table for the correlation coefficient (R^2^), it can be concluded that the experimental data fit the pseudo-second-order model more than the pseudo-first-order model, suggesting chemical adsorption of cyanide species on the surface of nanoparticles [43].

On the other hand, Figure 10 shows the fit of the experimental data to the intraparticle diffusion model. From this figure, the adsorption of total cyanide on the nanoparticles occurs conceptually in two stages (linear regions), after which intrinsic adsorption occurs, either by chemical binding on the active sites on the nanoparticles or by physical processes. The initial stage of fast speed that is observed in Figure 10 could be described as a process of diffusion of cyanide particles through the stationary film that surrounds each adsorbent nanoparticle; therefore, it corresponds to the mass transfer from the aqueous solution to the surface of the adsorbent. In contrast, the slow-rate second stage corresponds to intraparticle mass transfer and describes the process of diffusion of cyanide particles through the pores of adsorbent nanoparticles. The linear regression analysis for the diffusion kinetic models is displayed in Table 4. From the relatively high values of A reported in this table, it is suggested that surface adsorption could be the rate-limiting step for all synthesized nanoparticles [44]. 

### 4.4. Photodegradation Studies

#### 4.4.1. Kinetics of Cyanide Photodegradation

Regarding the photocatalytic behavior of TO, La/TO, Ce/TO, and Eu/TO, in this study, it was possible to demonstrate that these nanoparticles achieved effective removal of total cyanide in the aqueous solution. Possibly, this occurred because the doping of TiO_2_ with lanthanide elements La, Ce, and Eu not only provided greater quantity of active centers for adsorption of cyanide species but also the presence of these lanthanide elements on the surface of TiO_2_ promoted decrease in bandgap energy, which definitely enhanced the photoactivity of the doped nanoparticles. It is widely known that magnitude of bandgap energy is fundamental in photoactivity of semiconductors since it determines the recombination rate of the electron/hole pairs (e^−^/h^+^). Moreover, the recombination rate may depend on other factors, such as carrier concentration, carrier mobility, and semiconductor structure [81]. Therefore, the evidence from this study suggests that the presence of La, Ce, and Eu ions on the TiO_2_ surface enhanced the photoactivity of this semiconductor, probably by modifying the recombination rate of the pair (e^−^/h^+^). In addition, in this study, it was shown that the nanoparticles doped with La/TO, Ce/TO, and Eu/TO had lower bandgap energy than TO, so it is suggested that, given the smaller separation between the valence bands (VB) and conduction (CB), doped nanoparticles could easily transfer photoinduced electrons from the bulk to the surface and be more active than TO nanoparticles under simulated solar light [92].

The photocatalytic route involving the doped nanoparticles (La/TO, Ce/TO, and Eu/TO) begins with electronic excitation under simulated solar light. The photoexcited electrons (e^−^) are transferred from the VB ($e_{VB}^{-}$) to the CB ($e_{CB}^{-}$), leaving holes (h^+^) in the VB ($h_{VB}^{+}$) of the photocatalyst (Equation (21)). Due to this electronic transfer, electron/hole pairs (e^−^/h^+^) are photogenerated that migrate to the surface of the photocatalyst to react directly with the adsorbed species, such as H_2_O (${(H}_{2}{O)}_{ads}$), OH^−^ (${OH}_{ads}^{-}$), O_2_ (${\left(O_{2}\right)}_{ads}$), and other molecules. These (e^−^/h^+^) pairs can also recombine immediately after their formation (Equation (22)). On the other hand, the photogenerated holes (h^+^) in the VB of TiO_2_ promote oxidation of both adsorbed water molecules and hydroxyl ions to produce highly reactive hydroxyl radicals (Equations (23) and (24)). Furthermore, the holes (h^+^) can migrate towards the surface of the photocatalyst to create more reactive radicals and oxidize the molecules adsorbed on the surface. Lanthanide ions (Ln^n+^) have empty 5d orbitals, so they can trap photoexcited electrons in the CB of TiO_2_ (Equation (25)); however, these electrons are very unstable, so they are quickly transferred to oxygenated molecules adsorbed on the surface of the photocatalyst. Due to this transfer, radical superoxide anions (^•^O_2_^−^) and hydroxyl radicals (^•^OH) are generated through a series of sequential reactions (Equations (26)–(29)). The following reactions suggest the likely pathway for ROS photogeneration on the surface of Ln^(n+)^/TiO_2_ (Ln^(n+)^ = La^(3+)^, Ce ^(3+/4+)^, or Eu^(3+)^) [73,89]:

(21) $${Ln}^{n+}/TiO_{2}\overset{hv}{\to }{Ln}^{n+}/{TiO}_{2}+e_{CB}^{-}+h_{VB}^{+}$$

(22) $$e_{CB}^{-}+h_{VB}^{+}\to heat$$

(23) $${(H}_{2}{O)}_{ads}+h_{VB}^{+}⇌{\left(H^{+}+{OH}^{-}\right)}_{ads}+h_{VB}^{+}\to {OH}_{ads}^{⦁}$$

(24) $${OH}_{ads}^{-}+h_{VB}^{+}\to {OH}_{ads}^{⦁}$$

(25) $${Ln}^{\left(n+\right)}+e_{CB}^{-}\to {Ln}^{\left(n+\right)-1}$$

(26) $${Ln}^{\left(n+\right)-1}+{\left(O_{2}\right)}_{ads}\to {Ln}^{\left(n+\right)}+O_{2}^{⦁-}$$

(27) $$O_{2}^{⦁-}+H^{+}\to {HO}_{2}^{⦁}$$

(28) $${2HO}_{2}^{⦁}\to H_{2}O_{2}+O_{2}$$

(29) $$H_{2}O_{2}+e_{CB}^{-}\to {OH}^{⦁}+{OH}^{-}$$

Cyanide photodegradation occurs at the surface of the photocatalyst as a complex process involving transfer of multiple electrons and protons from the catalyst to cyanide species. Therefore, one of the key steps in photocatalytic degradation of cyanide is adsorption of cyanide ions on the surface of the photocatalyst [93].

Photocatalytic degradation of cyanide involves various reactive oxygen species (ROS), such as hydroxyl radicals (^•^OH), superoxide radicals (^•^O_2_^−^), and singlet oxygen (^1^O_2_), among others. As is well-known, the hydroxyl radical (^•^OH) is the most reactive species and can oxidize a wide range of contaminants, including cyanide. Oxidation of cyanide by hydroxyl radicals (^•^OH) can proceed through several pathways. In the biocatalytic pathway, the hydroxyl radical attacks the carbon atom of the cyanide ion to form formamide (NH_2_CHO), which can then react with the hydroxyl radicals to form formic acid (HCOOH) and ammonia (NH_3_) [94]. 

In the photocatalytic pathway, the hydroxyl radical attacks the nitrogen atom of the cyanide ion to form cyanate (CNO^−^) (Equation (30)), which can be oxidized to produce nitrogen gas, carbon dioxide gas, nitrate, or nitrite, and bicarbonate (Equations (31)–(34)) [95,96,97,98]. It is worth mentioning that hydrolysis of cyanate to bicarbonate and ammonium ions (Equation (35)) is more favored under acidic conditions (pH < 7) [99]. On the other hand, direct oxidation of cyanide can also occur if the molecule reacts directly with the photogenerated holes [79].

(30) $${CN}^{-}+{2\;OH}_{ads}^{⦁}\to {CNO}^{-}+H_{2}O$$

(31) OCN−+OHads⦁→CO2(g)+12N2(g)+H+

(32) OCN−+3 OHads⦁→HCO3−+12N2(g)+H2O

(33) $${OCN}^{-}+{6\;OH}_{ads}^{⦁}\to {HCO}_{3}^{-}+{NO}_{2}^{-}+H^{+}+{2H}_{2}O$$

(34) $${OCN}^{-}+{8\;OH}_{ads}^{⦁}\to {HCO}_{3}^{-}+{NO}_{3}^{-}+H^{+}+{3H}_{2}O$$

(35) $${OCN}^{-}+H^{+}+{2\;H}_{2}O\to {HCO}_{3}^{-}+{NH}_{4}^{+}$$

In addition to oxidation of cyanide by hydroxyl radicals, electrons in the conduction band of the photocatalyst may also participate in the degradation process. Electrons can reduce oxidized species (CNO^−^, CO_2_, N_2_) to less harmful and more stable forms, such as formate (HCOO^−^) or bicarbonate (HCO_3_^−^). The reduction process is driven by transfer of electrons from the photocatalyst to the oxidized species [94].

In summary, photocatalytic cyanide degradation is a process that can reduce cyanide toxicity in wastewater by generating reactive species through light absorption by a photocatalyst. The photodegradation mechanism of cyanide in an aqueous solution involves a series of reactions that depend on the type of photocatalyst used and the reaction conditions, including the initial cyanide concentration, light intensity, and the presence of other contaminants in the solution [91]. Although there are many factors that can affect the effectiveness of the cyanide photocatalysis process, the process has great potential as a treatment strategy for cyanide-contaminated wastewater.

#### 4.4.2. Effect of the Photogenerated Radicals

As shown in Figure 12, the efficiency of nanoparticles for photodegradation of cyanide species decreased with incorporation of i-POH and EtOH. This probably occurs due to quenching of h^+^ and ^•^OH, suggesting that these play a crucial role in photocatalytic degradation of cyanide species. However, Figure 12 did not show a significant effect on efficiency of photodegradation using p-BQ, probably because the presence of the radical ^•^O_2_^−^ is not essential for photocatalytic degradation of cyanide species under the conditions evaluated in this study. These results are in agreement with those informed by other authors [100]. 

### 4.5. Total Efficiency and Reuse of Nanoparticles

In this study, it was shown that photodegradation of cyanide species is more efficient than adsorption of these species under the test conditions (Figure 13). As is known, adsorption is a process that can be affected by a series of parameters, such as sorbent properties, sorbate properties, solution conditions, among others. In fact, in this study, it was shown that the pH of the solution had an important effect on adsorption capacity of cyanide species on the surface of nanoparticles. Thus, Figure 6 shows that the maximum adsorption capacity of cyanide species occurs at pH values > 9. However, since the adsorption tests were performed at pH = 7, it is possible that the relatively low adsorption percentages shown in Figure 13 were due to the limited electrostatic attraction between cyanide species (pKa = 9.4) and nanoparticle surface (pH_ZPC_ = 7). Although adsorption of cyanide species was limited by the pH of the solution, in this study, it was also possible to demonstrate that incorporation of lanthanide elements, particularly lanthanum, in the TiO_2_ structure is a good alternative to improve adsorption of these species on the semiconductor surface. Possibly, this was because incorporation of lanthanide elements as dopants enables generation of Lewis acid sites on the TiO_2_ surface, which undoubtedly contributes to improving the adsorption capacity of a semiconductor. 

Likewise, in this study, it was demonstrated that the combination of the adsorption and photocatalysis processes enables improvement in the efficiency of removal of cyanide species from aqueous solutions. This is probably because the cyanide species that first adsorbed and accumulated on the nanoparticle surface at the beginning of photocatalytic degradation are the ones that first degraded under simulated solar light. Consequently, the constant migration and successive photocatalytic oxidation on the surface of the nanoparticles certainly contribute to improving the removal efficiency at the solid–liquid interface. This is due to generation of a concentration gradient, which acts as the main driving force of the removal process of cyanide species from an aqueous solution. From these results, it is suggested that, in this study, removal of cyanide species is due to the cooperative effect between both adsorption and photocatalysis processes. Thus, the synthesized nanoparticles are effective for removal of cyanide from aqueous systems because they have coupled “adsorption–photodegradation” performances [88].

On the other hand, it is well-known that both the useful life of a material and its potential applications are closely related to its structural and chemical stability. In this study, an experiment was designed to evaluate reuse of synthesized nanoparticles in order to estimate their effectiveness after five cycles of consecutive use. The results of this experiment are shown in Figure 14. From this figure, it could be inferred that percentage of cyanide removal decreases with each treatment cycle. However, after five consecutive cycles, loss of cyanide removal capacity of the nanoparticles did not exceed 20% on average. In fact, in this study, it was found that La/TO nanoparticles had a lower loss of effectiveness (15.3%) at the end of the fifth reuse cycle compared to Ce/TO (16.6%) and Eu/TO (17.8%) nanoparticles. The loss in effectiveness was probably due to chemical adsorption of cyanide species on the surface of the nanoparticles since the possible formation of covalent bonds and complexes could decrease the availability of active sites on the surface. At the end of the fifth cycle, the chemical composition of the nanoparticles was verified by ICP analysis, confirming that there was no lanthanide leakage into the solution. Consequently, the synthesized nanoparticles (TO, La/TO, Ce/TO, and Eu/TO) are stable and maintain adequate activity until the fifth treatment cycle, being able to effectively remove cyanide species in aqueous solution.

Finally, Table 5 compares the maximum adsorption capacity (mg g^−1^) of the present nanoparticles and some adsorbents used for cyanide removal from aqueous solutions.

Likewise, Table 6 compares the photodegradation efficiency (%) of the present nanoparticles and some photocatalysts used for cyanide removal from aqueous solutions.

As can be seen in Table 5 and Table 6, among the nanoparticles synthesized in this study, La/TiO_2_ showed the highest capacity for adsorption and photodegradation of cyanide species in aqueous solutions. However, compared to other materials based on metal oxides (ZnO, NiO, Fe_2_O_3_) reported in the literature, La/TiO_2_ (q_max_ = 54.96 mg g^−1^) has a lower adsorption capacity for cyanide species under the tested conditions, although it turned out to be a better adsorbent material for cyanide species than certain zeolitic materials and biochar. On the other hand, La/TiO_2_ also proved to be more efficient for cyanide photodegradation than the other nanoparticles synthesized in this study. Likewise, La/TiO_2_ proved to be the same and even more efficient for cyanide photodegradation than other TiO_2_-based photocatalytic materials recently reported in the literature, reaching an efficiency of 97% after 90 min of reaction, with a catalyst load of 0.2 g L^−1^ and a cyanide concentration in the solution of 20 mg L^−1^.

Based on the adsorption and photodegradation results obtained in this study and comparing them with those reported in the literature cited above, it is suggested that La/TO, Ce/TO, Eu/TO, and TO nanoparticles, in that order, are effective for removal of cyanide species from aqueous solutions. Furthermore, the evidence from this study suggests that the removal of cyanide species was governed by a combination of electrostatic interactions, complex formation by coordinate covalent bonds, and photo-oxidation on the surface of the synthesized nanoparticles when the reaction systems were exposed to simulated solar light.

## 5. Conclusions

The evidence from this study indicates that the adsorption and photodegradation capacity of the nanoparticles synthesized in the present study (TiO_2_, La/TiO_2_, Ce/TiO_2_, and Eu/TiO_2_) were influenced by several factors. Some of these were dependent on material properties (chemical composition, bandgap energy, crystal structure, morphology, particle size, specific surface area, and pH_PZC_), as well as the operating conditions (initial concentration of adsorbate, contact time, system temperature, and pH of the solution) of the removal processes used. These results are in agreement with those reported by other authors [120]. Investigation of these parameters enabled us to obtain valuable information on the mechanisms of adsorption and photocatalysis, which was fundamental to determining the best operational conditions for efficient removal of cyanide species from aqueous solutions at neutral pH. Likewise, synergistic coupling of the adsorption and photocatalysis processes enabled significant improvement in the capacity of the TiO_2_ semiconductor for effective removal of cyanide species from aqueous solutions.

Our results showed that doping of the TiO_2_ semiconductor with lanthanide elements La, Ce, and Eu represents a promising alternative to improve the adsorption capacity of the TiO_2_ semiconductor and extend its photoresponse to the visible light region. Among the doped nanoparticles, La/TO was more efficient than Ce/TO and Eu/TO for total cyanide removal, probably because the La/TO nanoparticles had a higher specific surface area (126 m^2^ g^−1^) in comparison with the specific surface area of Ce/TO (104 m^2^ g^−1^) and Eu/TO (96 m^2^ g^−1^). Regarding cyanide photodegradation, it was also influenced by type of doping lanthanide ion. La/TO nanoparticles were slightly more effective under simulated solar light than Ce/TO and Eu/TO nanoparticles, possibly because the La/TO bandgap energy (E_g_ = 3.10 eV) was lower than that of Ce/TO (E_g_ = 3.14 eV) and Eu/TO (E_g_ = 3.16 eV), which is essential for absorption of light and generation of pairs of electrons and voids for photodegradation of pollutants. On the other hand, reuse of doped nanoparticles was also affected by type of lanthanide element. In this study, it was found that La/TO nanoparticles had lower loss of effectiveness (15.3%) at the end of the fifth reuse cycle compared to Ce/TO (16.6%) and Eu/TO (17.8%) nanoparticles, probably due to the smaller particle size of La/TO compared to Ce/TO and Eu/TO, which could provide greater structural stability to the photocatalyst in aqueous reaction systems. Therefore, the evidence from this comparative study suggests that each doping element has an intrinsic effect on the properties of TiO_2_, making it essential to understand these effects in the design of effective materials for wastewater treatment. 

In summary, this study provides information on the potential capacity of doping TiO_2_ nanoparticles with lanthanides (La, Ce, and Eu) as an innovative and effective approach to improve the adsorption and photocatalysis properties of a semiconductor. In addition, it highlights the synergistic effect of combining both techniques, adsorption and photocatalysis, to achieve complete and efficient removal of cyanide in wastewater, which is valuable for development of new technologies that contribute to treatment and recovery of hydric resources. Finally, it supports use of solar energy as a lighting source during removal of cyanide in aqueous systems, contributing to generation of beneficial effects in terms of environmental impact, energy efficiency, and remediation costs, thus promoting sustainable practices.

## Figures and Tables

**Table 1:** Effect of nanoparticles compositions on their cyanide adsorption capacity.

Nanoparticles Composition	HSD Tukey *	Duncan *
	q_e_ (mg g^−1^)	q_e_ (mg g^−1^)
La/TO (1.0%)	1.18 ± 0.07 ^a^	1.18 ± 0.07 ^a^
Ce/TO (1.0%)	2.24 ± 0.11 ^b^	2.24 ± 0.11 ^b^
La/TO (0.5%)	2.43 ± 0.09 ^b^	2.43 ± 0.09 ^b^
Eu/TO (1.0%)	3.07 ± 0.02 ^c^	3.07 ± 0.02 ^c^
Ce/TO (0.5%)	3.22 ± 0.10 ^c^	3.22 ± 0.10 ^c^
La/TO (2.0%)	3.47 ± 0.23 ^c^	3.47 ± 0.23 ^c^
Eu/TO (0.5%)	4.28 ± 0.29 ^d^	4.28 ± 0.29 ^d^
Ce/TO (2.0%)	4.79 ± 0.31 ^d^	4.79 ± 0.31 ^e^
Eu/TO (2.0%)	5.73 ± 0.16 ^e^	5.73 ± 0.16 ^f^
TO	6.16 ± 0.07 ^e^	6.16 ± 0.07 ^g^
*p*-value	<0.001	<0.001

Different letters (a–g) indicate different groups with a high level of statistical significance (*p* < 0.01). The means for the groups in the homogeneous subsets are displayed. * Use the sample size of the harmonic mean = 3.0.

**Table 2:** Isotherm parameters for cyanide sorption on nanoparticles at different temperatures.

Isotherm Parameters		293.15 K				298.15 K				303.15 K			
		TO	La/TO	Ce/TO	Eu/TO	TO	La/TO	Ce/TO	Eu/TO	TO	La/TO	Ce/TO	Eu/TO
Langmuir	q_max_ (mg g^−1^)	46.48 (±4.10)	54.96 (±3.52)	51.39 (±2.11)	49.25 (±1.52)	51.13 (±2.56)	60.46 (±2.42)	56.53 (±3.18)	54.17 (±1.24)	55.78 (±2.32)	68.95 (±2.76)	61.67 (±3.01)	59.10 (±1.43)
	K_L_ (L mg^−1^)	0.17 (±0.04)	0.22 (±0.04)	0.26 (±0.04)	0.31 (±0.03)	0.18 (±0.04)	0.24 (±0.03)	0.28 (±0.04)	0.33 (±0.04)	0.19 (±0.04)	0.27 (±0.03)	0.31 (±0.04)	0.37 (±0.03)
	R_L_	0.23	0.18	0.16	0.14	0.22	0.17	0.15	0.13	0.21	0.16	0.14	0.12
	χ^2^	2.89	3.21	3.97	2.45	2.67	3.30	3.78	2.36	3.64	2.41	2.81	3.65
	R^2^	0.95	0.97	0.98	0.99	0.97	0.96	0.99	0.98	0.96	0.98	0.97	0.99
Freundlich	K_F_ (L mg^−1^)	9.35 (±2.24)	13.02 (±2.59)	13.65 (±2.25)	14.76 (±1.94)	10.28 (±2.62)	14.32 (±2.63)	15.02 (±2.41)	16.24 (±1.78)	11.22 (±2.12)	15.62 (±2.85)	16.38 (±2.34)	17.71 (±1.36)
	n	2.25 (±0.44)	2.40 (±0.44)	2.56 (±0.41)	2.80 (±0.39)	2.48 (±0.47)	2.64 (±0.41)	2.82 (±0.44)	3.07 (±0.37)	2.70 (±0.41)	2.88 (±0.40)	3.08 (±0.39)	3.35 (±0.35)
	1/n	0.44	0.42	0.39	0.36	0.40	0.38	0.35	0.33	0.37	0.35	0.33	0.30
	χ^2^	3.95	3.15	2.96	2.70	4.20	3.02	3.12	2.81	3.56	3.42	3.15	2.15
	R^2^	0.84	0.86	0.89	0.92	0.86	0.89	0.90	0.94	0.87	0.90	0.88	0.92

**Table 3:** Thermodynamic parameters of cyanide adsorption onto nanoparticles.

NPs	Temperature (K)	ln k_C_	∆G° (kJ mol^−1^)	∆H° (kJ mol^−1^)	∆S° (kJ mol^−1^ K^−1^)
TO	293.15	12.45	−30.34	9.74	0.14
	298.15	12.50	−30.99		
	303.15	12.58	−31.70		
La/TO	293.15	12.72	−31.01	13.43	0.15
	298.15	12.80	−31.72		
	303.15	12.91	−32.53		
Ce/TO	293.15	12.89	−31.42	11.33	0.15
	298.15	12.95	−32.10		
	303.15	13.04	−32.87		
Eu/TO	293.15	13.05	−31.80	12.94	0.15
	298.15	13.12	−35.52		
	303.15	13.22	−33.32		

**Table 4:** Kinetic parameters for cyanide sorption on nanoparticles.

Kinetic Parameters		TO	La/TO	Ce/TO	Eu/TO
Pseudo-first-order	q_max_ (mg g^−1^)	136.53 (±2.51)	143.85 (±1.69)	141.83 (±1.81)	137.82 (±2.94)
	*k*_1_ (L mg^−1^)	0.04 (±2.29 × 10^−3^)	0.03 (±1.02 × 10^−3^)	0.03 (±1.56 × 10^−3^)	0.03 (±2.45 × 10^−3^)
	χ^2^	8.94	8.48	7.69	7.13
	R^2^	0.95	0.99	0.99	0.99
Pseudo-second-order	q_max_ (mg g^−1^)	152.22 (±3.67)	175.08 (±6.36)	165.40 (±2.45)	160.69 (±3.99)
	*k*_2_ (L mg^−1^)	3.19 × 10^−4^ (±2.75 × 10^−5^)	1.55 × 10^−4^ (±2.41 × 10^−5^)	2.37 × 10^−4^ (±1.70 × 10^−5^)	2.37 × 10^−4^ (±2.81 × 10^−5^)
	χ^2^	2.82	3.03	3.67	2.64
	R^2^	0.99	1.00	1.00	0.99
Intraparticle diffusion	*k*_3_ (mg g^−1^ min^−1/2^)	9.94 (±0.27)	10.80 (±0.17)	10.40 (±0.21)	9.89 (±0.16)
	*A*	8.09 (±1.25)	9.99 (±1.05)	8.37 (±1.15)	9.12 (±1.17)
	R^2^	0.91	0.92	0.90	0.92
External-film diffusion	D*f* (m^2^ min^−1^)	1.14 × 10^−11^	1.14 × 10^−11^	1.25 × 10^−11^	1.10 × 10^−11^
	R^2^	0.98	0.99	0.98	0.99
Internal-pore diffusion	D*p* (m^2^ min^−1^)	1.40 × 10^−17^	1.42 × 10^−17^	1.60 × 10^−17^	1.40 × 10^−17^
	R^2^	0.97	0.97	0.96	0.97

**Table 5:** Comparison of adsorption capacity (mg g^−1^) of various materials for cyanide removal.

Adsorbent	q_max_ (mg g^−1^)	Isotherm Model	Kinetic Model	Reference
ZnO	275.00	Langmuir	Pseudo-second-order	[43]
NiO	185.00	Langmuir	Pseudo-first-order	[43]
ZnO-NiO	320.00	Langmuir	Pseudo-second-order	[43]
LTA zeolite modified with HDMTMAB	24.09	Langmuir	-	[44]
Activated Carbon (AC)	78.10	Redlich-Peterson	Pseudo-second-order	[48]
Fe_2_O_3_/AC	86.20	Redlich-Peterson	Pseudo-second-order	[48]
TiO_2_/AC	90.90	Redlich-Peterson	Pseudo-second-order	[48]
ZnO/AC	91.70	Redlich-Peterson	Pseudo-second-order	[48]
TiO_2_/Fe_2_O_3_/AC	96.20	Langmuir	Pseudo-second-order	[48]
ZnO/Fe_2_O_3_/AC	101.00	Langmuir	Pseudo-second-order	[48]
Clay-K	253.98	-	Pseudo-second-order	[91]
TiO_2_/Fe_2_O_3_	124.87	-	Pseudo-second-order	[91]
Fe-MFI zeolite	33.98	Langmuir	Pseudo-second-order	[101]
Activated Periwinkle Shell Carbon (APSC)	2.85	Langmuir	Pseudo-second-order	[102]
SiO_2_/TiO_2_	39.79	Temkin	Pseudo-second-order	[103]
Activated Carbon (AC)	1.66	Freundlich	Pseudo-second-order	[104]
LDH loaded MMB	80	Langmuir	-	[105]
Corncob biochar	2.57	Langmuir	-	[106]
TiO_2_	46.48	Langmuir	Pseudo-second-order	In this study
La/TiO_2_	54.96	Langmuir	Pseudo-second-order	In this study
Ce/TiO_2_	51.39	Langmuir	Pseudo-second-order	In this study
Eu/TiO_2_	49.25	Langmuir	Pseudo-second-order	In this study

**Table 6:** Comparison of photodegradation efficiency (%) of various materials for cyanide removal.

Material	[CN] (mg L^−1^]	[Catalyst] (g L^−1^]	Time (min)	Efficiency (%)	Reference
TiO_2_/Fe_2_O_3_/zeolite	200	1.4	160	89	[48]
TiO_2_/Fe_2_O_3_/PAC	300	1.4	170	97	[48]
Blast furnace sludge (BFS)	750	2.0	120	97	[91]
Cts-Ag	71.6	2.5	180	98	[100]
SiO_2_/TiO_2_	61.54	3.5	30	96	[103]
Fe^2+^	10	0.14	30	86	[107]
TiO_2_	30	0.05	60	72	[108]
Co/TiO_2_/SiO_2_	100	2.0	60	55	[109]
TiO_2_/SiO_2_	100	1.7	180	93	[110]
Ce/ZnO	250	4.0	180	84	[111]
Degussa P25 TiO_2_	13.2	0.1	60	73	[112]
Cu(II)-cryptate, complex 1	6.07	0.5–1.0	180	77	[113]
S–TiO_2_@rGO-FeTCPP	100	1.6	120	75	[114]
N-rGO-ZnO-CoPc(COOH)_8_	25	2.0	120	91	[115]
TiO_2_/ZSM-5	71.55	2.5	240	94	[116]
Me (Fe, Mn)-N/TiO_2_/SiO_2_	75	2.5	120	97	[117]
Carbon/nano-TiO_2_	61.53	3.0	180	98	[118]
rGO/TiO_2_ P25	50	1.0	180	100	[119]
TiO_2_	20	0.2	90	84	In this study
La/TiO_2_	20	0.2	90	97	In this study
Ce/TiO_2_	20	0.2	90	89	In this study
Eu/TiO_2_	20	0.2	90	86	In this study

## Data Availability

Data are contained within the article.
